# Flexural Response of Concrete Specimen Retrofitted with PU Grout Material: Experimental and Numerical Modeling

**DOI:** 10.3390/polym15204114

**Published:** 2023-10-17

**Authors:** Sadi Ibrahim Haruna, Yasser E. Ibrahim, Zhu Han, Abdulwarith Ibrahim Bibi Farouk

**Affiliations:** 1 Engineering Management Department, College of Engineering, Prince Sultan University, Riyadh 11586, Saudi Arabia; 2 School of Civil Engineering, Tianjin University, Tianjin 300072, China; hanzhu2000@tju.edu.cn (Z.H.); aifg1986@tju.edu.cn (A.I.B.F.)

**Keywords:** concrete, polyurethane, finite element analysis, polyurethane grout material, flexural strength

## Abstract

Polyurethane (PU) composite is increasingly used as a repair material for civil engineering infrastructure, including runway, road pavement, and buildings. Evaluation of polyurethane grouting (PUG) material is critical to achieve a desirable maintenance effect. This study aims to evaluate the flexural behavior of normal concrete repaired with polyurethane grout (NC-PUG) under a three-point bending test. A finite element (FE) model was developed to simulate the flexural response of the NC-PUG specimens. The equivalent principle response of the NC-PUG was analyzed through a three-dimensional finite element model (3D FEM). The NC and PUG properties were simulated using stress–strain relations acquired from compressive and tensile tests. The overlaid PUG material was prepared by mixing PU and quartz sand and overlayed on the either top or bottom surface of the concrete beam. Two different overlaid thicknesses were adopted, including 5 mm and 10 mm. The composite NC-PUG specimens were formed by casting a PUG material using different overlaid thicknesses and configurations. The reference specimen showed the highest average ultimate flexural stress of 5.56 MPa ± 2.57% at a 95% confidence interval with a corresponding midspan deflection of 0.49 mm ± 13.60%. However, due to the strengthened effect of the PUG layer, the deflection of the composite specimen was significantly improved. The concrete specimens retrofitted at the top surface demonstrated a typical linear pattern from the initial loading stage until the complete failure of the specimen. Moreover, the concrete specimens retrofitted at the bottom surface exhibit two deformation regions before the complete failure. The FE analysis showed good agreement between the numerical model and the experimental test result. The numerical model accurately predicted the flexural strength of the NC-PUG beam, slightly underestimating Ke by 4% and overestimating the ultimate flexural stress by 3%.

## 1. Introduction

**Figure 1:**
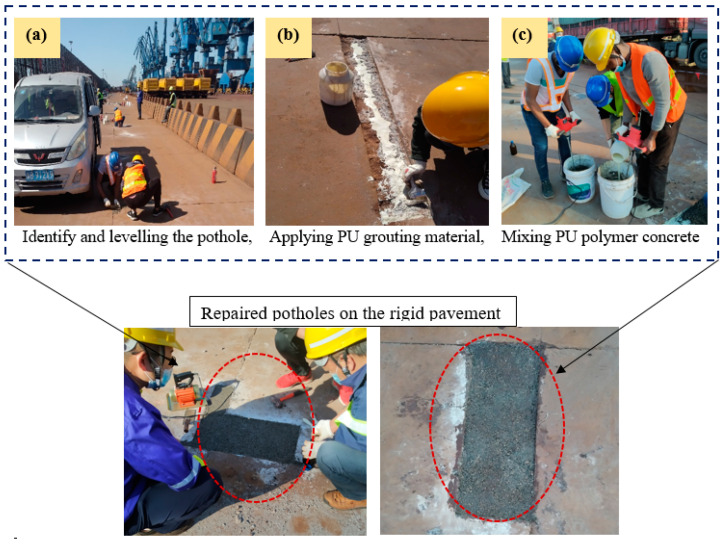
Engineering application of polyurethane-based polymer composites.

Repair and protective techniques are applied to civil engineering infrastructure, including reinforced concrete structures, roads, and runways, by exposing damaged sections and changing them with cement-based composites [1,2,3,4,5]. Due to the cracking of the replaced materials and further penetration of degradation substances into concrete structures, the functions of the repaired section may deteriorate again [6,7]. Water can be an essential carrier for aggressive, penetrating substances [6]. Thus, efficient means to restrict water penetration into repaired areas and underlying concrete structures is critical for preserving their high resilience and extended lifetimes. Alternative materials of polymeric resins, including methyl methacrylate (MMA), epoxy resins, furan resins, polyurethane resins, urea formaldehyde, and unsaturated polyester resins, are available to maintain concrete structure effectively. Moreover, latex (polymer suspension in water), powder, and resin (liquid form) are all common types of polymer modifiers. Similarly, there are polymer modifiers, which include styrene butadiene rubber (SBR) emulsion, ethylene vinyl acetate (EVA), polyacrylate (PAE) emulsion, epoxy resin (EP), polyvinyl alcohol vinyl acetate, ethylene vinyl acetate, and acrylic acid [8,9,10,11,12]. PU is a hard polymer with good wear-resistance characteristics [13]. The PU-cement-based composite was reportedly employed in retrofitting structures after a seismic event because of its high bending and low compressive strength reduction [14]. Polyurethane gout materials have been used in several repair projects, including highway crack treatment, high-speed railway track slab raising, emergency reinforcement of water conservation projects, and repair of rigid pavement [15,16,17,18], as depicted in Figure 1. Similarly, closed-cell one-component hydrophobic polyurethane foam can be used to stabilize expansive soil [19], whereas polyurethane grouting materials are employed for repair of road and runway facilities [20].

The numerical analytical method has been utilized to investigate the performance of concrete materials under static loadings, which is considered isotropic and homogenous for numerical simulation [21,22,23,24]. The numerical analysis was carried out using the finite element approach, demonstrating that the numerical analysis could be utilized as an evaluation tool for analyzing the different performance of the polyurethane-concrete composite [25,26,27,28]. Hala et al. [25] conducted an experimental study and numerical analysis on the ballistic resistance of high-performance fiber-reinforced concrete panels coated with polyurethane materials. The numerical models adequately predict the ballistic strength of the panels under independent ballistics tests. Sing et al. [29] evaluated the compression, tension, and flexural properties of four epoxy grouts and developed a finite element model to simulate composite repaired pipes. The result showed good agreement between the FE models and the experimental test result with a margin error of less than 10%. It was discovered that by modifying the infill parameters in the finite element model to simulate the usage of different infill materials for the repair, a 4–8% increase in burst pressure can be produced. Shigang et al. [30] performed numerical simulations of the polyurethane polymer concrete specimens in compression under different strain rates utilizing explicit numerical methods based on LS-DYNA codes. The failure factors of polymer concrete at the mesoscale level were numerically analyzed. The result indicated that the novel dynamic properties of the material attributed to the damage and failure mode of the interface and elastic/plastic properties of the polyurethane polymer composites. Chen et al. [31] conducted a long-term study and numerical simulation of PU foam insulation on concrete dams under extreme cold conditions. The FE analysis on the composite profiled sheet deck formed by applying polyurethane and polyvinyl chloride tubes was studied by [32]. Manjun et al. [28] established the FE model to simulate the shear failure process of polyurethane–bentonite composite specimens under variable angle shear test; the results indicated that the FE model result is consistent with the experimental result. Somarathna et al. [33] studied the dynamic mechanical properties of concrete retrofitted with polyurethane coating material subjected to quasi-static and dynamic loads simulated via a three-point bending test. The failure mechanism between the PU grout and concrete under the influence of moisture was investigated using digital image correlation [34]. Huang et al. [35] proposed a calculation technique to determine the deformation of precast concrete frame assembled with artificial controllable plastic hinges, and performed seismic analysis. The result showed that artificial controllable plastic hinges effectively reduced the base shear of the frame structure. The seismic performance of corrosion-damaged reinforced concrete columns strengthened with a bonded steel plate (BSP) and a high-performance ferrocement laminate (HPFL) was evaluated [36]. Zhang et al. [37] developed a numerical model and reliability-based analysis of the flexural strength of concrete beams reinforced with hybrid basalt fiber-reinforced polymer and steel rebars.

Studying the performance properties of composite concrete retrofitted with polyurethane grout under FE simulation requires considerable attention, as most previous studies paid attention to the experimental investigation, even though experimental studies were an appropriate means of understanding the structural response. Experimental studies are expensive, time-consuming, and unviable, especially for comprehensive or parametric studies. Thus, when accurately calibrated and validated, the FE analysis technique is an alternative way of investigating the structural responses. This study intends to investigate the effectiveness and capacity of the PU material prepared by combining bio-based polyurethane (castor oil) and quartz sand as a coating material for a concrete beam subjected to a three-point bending test and a developed FE simulation of the concrete–polyurethane grout (NC-PUG) under a flexural load. The equivalent principle response of the NC-PUG was analyzed through a three-dimensional finite element model (3D FEM). The NC and PUG properties were simulated using stress–strain relations acquired from compressive and tensile tests. The concrete damage plasticity model (CDPM) available in commercially available FE software ABAQUS 2021 [38] is utilized to model the response of normal concrete and polyurethane grout material.

## 2. Materials and Methods

### 2.1. Materials

**Figure 2:**
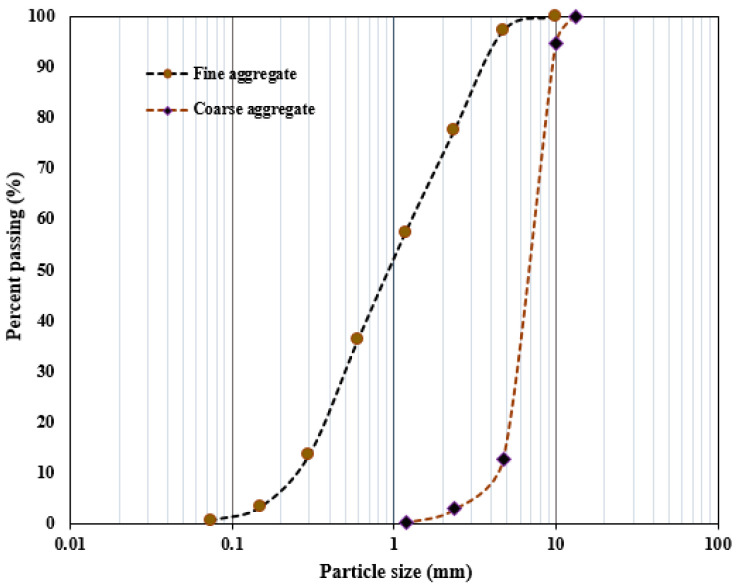
Grading curve of the aggregate materials.

Grade 42.5R ordinary Portland cement was used to produce a concrete mixture, and its chemical compositions are summarized in Table 1. The fine aggregate in this study was a river sand with 2650 kg/m^3^ density 2.63 fineness modulus. The coarse aggregate is the crushed natural stone with 10 mm aggregate size and 2.67 fineness modulus. Figure 2 presents the distribution of the aggregate sizes used. The quartz sand has particles the size of 0.5–1.0 mm and density of 1430 kg/m^3^. The desired workability of the concrete was maintained by adding polycarboxylate-based superplasticizer at 0.15% of the weight of cement to obtain required workability.

The PU binder was synthesized through a polymerization reaction between polyaryl polymethylene, isocyanate (PAPI), and castor oil, mixed at the mix ratio of 6:1, and placed in a container. A homogenous solution was produced after 2 min of rigorous mixing with hand mixer set at high speed [17,39,40]. Table 2 shows the physical and performance indexes of the PU binder.

### 2.2. Specimen Preparation

Table 3 shows the mix proportion for the preparation of concrete mixture and PU grouting materials. The NC mix was poured into beam molds with defined dimensions of 100 × 100 × 400 mm^3^. To obtain a satisfactory level of compaction, the cast specimens were put on the vibrating table. They were then left at room temperature for around 24 h before being removed from the mold. Furthermore, all the specimens were cured for 28 days prior to the test.

**Figure 3:**
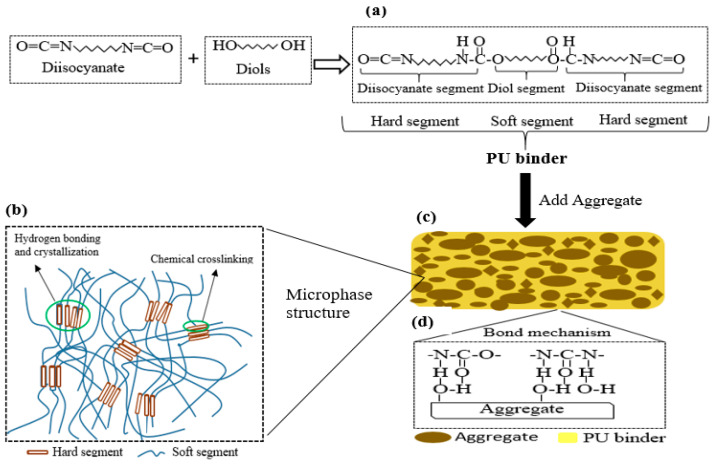
Systemic illustration of (**a**) production of PU binder, (**b**) PU molecular structure, (**c**) PUG geometric model, and (**d**) bond mechanism between PU binder and quartz sand.

**Figure 4:**
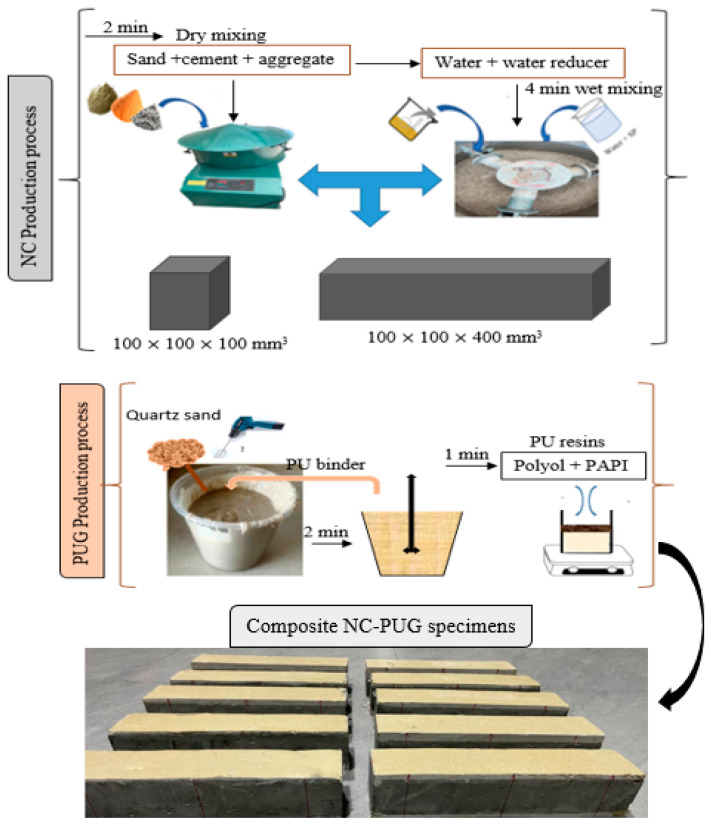
The graphical representation of the process of preparing NC-PUG composite.

After 28 days, all samples were air dried for seven days to make sure that all the surface moisture was completely dried before PU grouting was overlaid. The prepared PU grouting materials were synthesized by mixing quartz sand and PU binder using a mixing ratio of 1:0.5 in relation to weight. A homogenous mixture of quartz sand and PU binder was obtained by rigorous mixing of the two components using a hand mixer at high speed. Table 3 presents the preparation process of the PUG. The synthetic route toward producing the PUG binder and its microphase structure is shown in Figure 3a,b. Therefore, the PUG was cast either at the top and bottom or both surfaces of the concrete beam at 5 mm and 10 mm thicknesses, as indicated in Table 4. The NC-PUG composite configuration used to conduct flexural tests is shown in Table 4. The graphical representation process of preparing the composite beam and strengthened with PU grouting material is depicted in Figure 4.

### 2.3. Test Methodology

#### 2.3.1. NC Compressive Test

The NC cube compression test was conducted following 50081-2002 [41] using 100 cube specimens. The 20-ton loading capacity (WDW 200E) universal testing machine was used to test the NC compressive strength. Three samples were tested after 28 days of curation, and the average was considered as the strength of normal concrete.

#### 2.3.2. The NC-PUG Flexural Test

**Figure 5:**
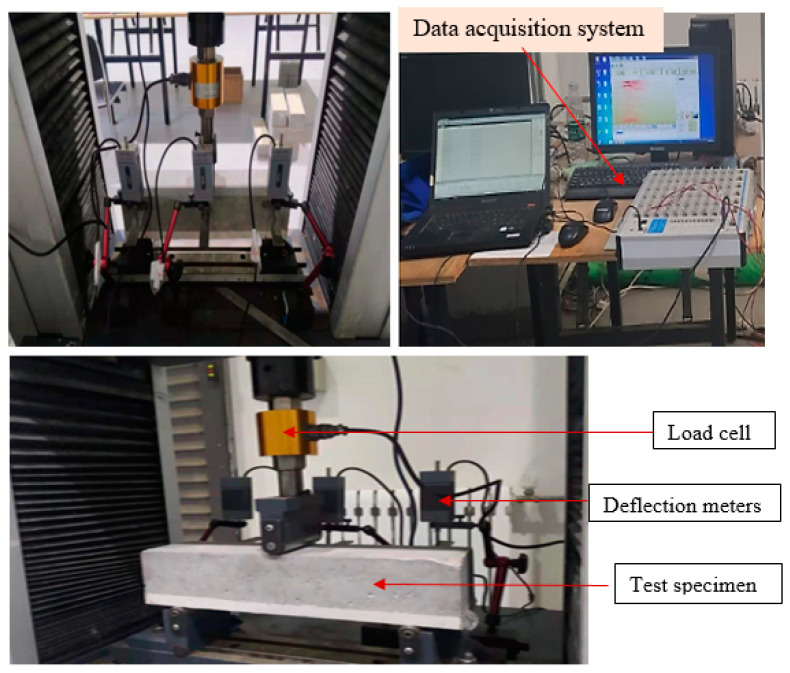
Experimental setup for flexural test.

Figure 5 presents the setup for the flexural strength test. The NC-PUG flexural behavior was tested according to the Chinese national standard GB/T 50081-2002 [41]. Using a 300 mm clear span loading and a 0.85 size reduction coefficient, a three-point bending method was used to calculate the flexural stress [41]. The UTM was set at 0.05 mm/min displacement-controlled loading and the specimens were loaded until complete specimen failure. The NC-PUG flexural strength was determined based on Equation (1). Moreover, three LVDTs were attached to the test specimen to monitor the deflection at midspan and two end supports, as illustrated in Figure 5. To add the load to the test sample, a load cell was coupled to the apparatus. The datasets for time, load, and displacement were simultaneously captured with a static data collecting system.

(1) $$f_{y}=\frac{Pl}{bh^{2}}\times 0.85$$

where *f*_y_ is the NC-PUG and the flexural strength (MPa); *P* represents the maximum applied load (KN); *l* represents the distance of the two supports (mm); and *h* and *b* represent the height and width of the beam section (mm), respectively.

## 3. Result and Discussion

### 3.1. Flexural Response of NC-PUG

**Figure 6:**
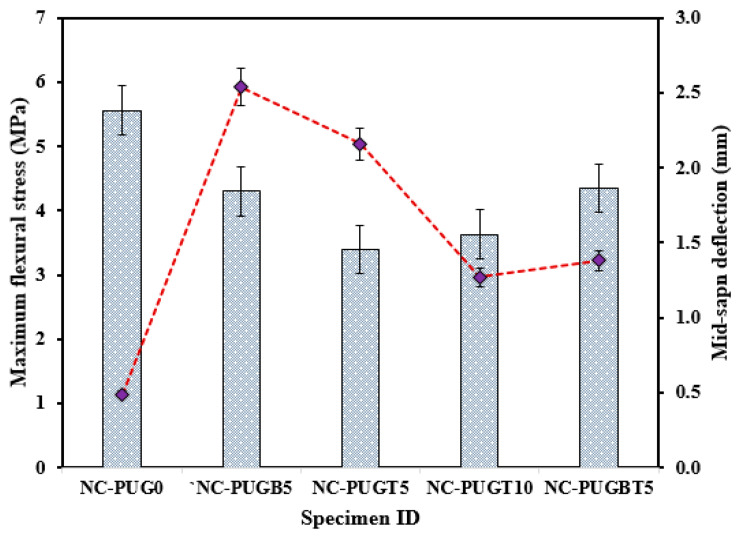
The flexural response of NC-PUG concrete.

Table 5 summarizes the response of the NC repaired with the PU grout material under flexural load. The average of the three samples tested under each testing condition was computed, and the flexural and deflection response was considered based on the 95% confidence level. As shown in Table 5, the control sample (NC-PUG0) reveals the highest average ultimate flexural stress of 5.56 MPa ± 2.57% against the concrete specimen retrofitted with the PUG grouting material; furthermore, a minimum mid-span deflection of 0.49 mm ± 13.60% was recorded for the control sample. However, the ultimate flexural stress of the specimens retrofitted with the PU grouting materials demonstrated reduced flexural strength with increasing deflection, as presented in Table 6. The flexural response is seen in Figure 6, showing the decreasing and increasing pattern of the NC-PUG composite due to the retrofitting effect of the PU grout material and casting configuration. The specimen repaired with a 5 mm thick PUG overlaid at the bottom surface (NC-PUGB5) and top-bottom surface (NC-PUGTB5) showed nearly the same ultimate flexural stress of 4.30 MPa ± 1.77% and 4.35 MPa ± 3.62%, respectively, which are lower than that of the reference specimen by 22.66% and 21.76%, respectively. The mid-span deflection of NC-PUGB5 is 2.19 mm ± 5.26%, and that of NC-PUGB5 is 1.40 mm ± 1.66%. The specimen retrofitted with 5 mm and 10 mm overlaid the PU grout material at the top surface exhibits the ultimate flexural stress of 3.39 MPa ± 6.80% and 3.63 MPa ± 3.72%, which are lower than the flexural strength of the specimen retrofitted at the bottom surface. This behavior is attributed to the specimen attaining a maximum carrying load and then failing. The concrete section no longer bears the applied load. At this stage, the overlaid PU grout sustained the applied load to some specific point before the ultimate failure of the NC-PUG composite, indicating the viscoelastic properties of polyurethane, which tend to make concrete more ductile and less brittle. Following the experimental research utilizing polyurethane demonstrates that PU is a very strain rate-sensitive elastomer, with a significant change in performance from rubbery to leathery in response to increased strain rates [42,43,44,45].

**Figure 7:**
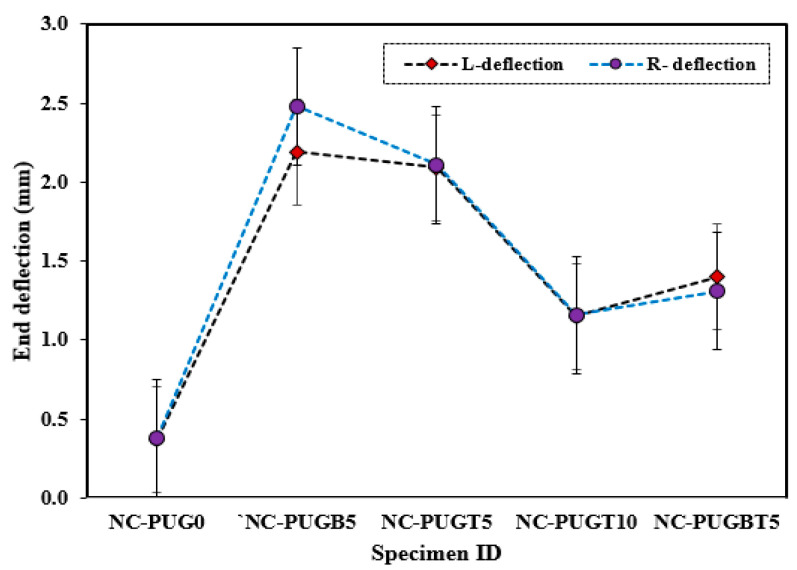
Deflection behavior at the two ends of the specimens.

Additionally, deflection due to the applied load was recorded at the two end supports, as shown in Figure 7. As shown in Figure 7, the deflection at the end support showed increasing and decreasing behavior according to the overlaid thickness and configuration. Reference specimens exhibit the lowest average deflection of 0.49 mm ± 13.60% and 0.38 mm ± 9.73% at the left and right support, respectively. This deflection is drastically increased due to the PU grouting effect, as exhibited in NC-PUGB5, which records an average deflection of 2.54 ± 2.62% and 2.48 ± 7.81% at the left and right support, respectively. The support deflection tends to decrease with a change in the PUG overlaid thickness and casting position. The support deflection of composite specimens with a 5 mm thick PUG cast at the top surface is reduced by 14.6% compared to the composite specimen cast with a 5 mm PUG overlaid thickness at the bottom surface. The support deflection decreased further in the specimen cast with a 10 mm thick PUG overlaid at the top surface. Hence, the record lowest recorded support deflection of 1.27 mm ± 10.55% and 1.16 mm ± 8.57%, respectively, was obtained. An improvement in the support deflection was observed in the NC-PUGBT5 specimen when compared to the NC-PUGT10 composite specimen. This result indicated that the deflection behavior of concrete can be improved with PUG grouting materials, and was more pronounced when the PUG overlaid was cast at the bottom surface. A related study by Somarathna et al. [33] reported that concrete specimens retrofitted externally reveal a higher strain during ultimate failure due to the elastomeric coating on the impact face under quasi-static testing.

### 3.2. Finite Element Modeling of NC-PUG Beam

The finite element modeling (FEM) and analysis (FEA) of the NC-PUG beam were carried out using the FE software ABAQUS 2021 [46]. FEM can help evaluate the properties of the concrete-to-polyurethane grout composite (NC-PUG). The details of the FEM, including the geometry, meshing, and boundary conditions, are presented in this section. This section presents the FE model development to evaluate the flexural responses of the NC-PUG beam.

### 3.3. Boundary Conditions, Loading Analysis, and Interaction

**Figure 8:**
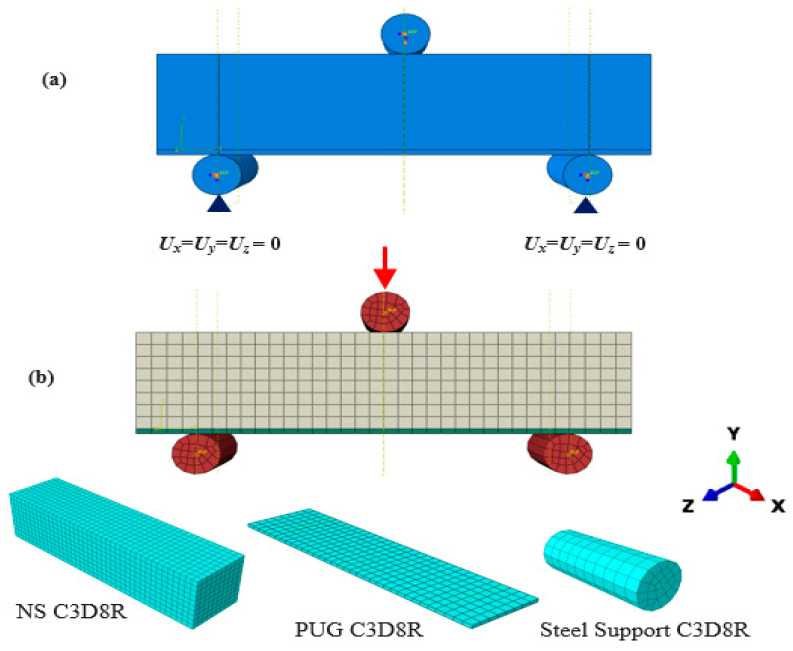
FE model of the NC-PUG beam. (**a**) Boundary condition; (**b**) meshing.

The specimens were fixed from the bottom steel support (Figure 8a). Displacement-controlled type of loading was applied to the simulation system. The NC-PUG contact was regarded for surface-to-surface relations. The firm contact without penetration was employed, and the shear characteristic was defined using the “penalty” function. The friction coefficient 0.4 between the NC substrate and PUG layer interface was considered. The NS surface was designed as the master surface, while the PUG surface was set as the slave surface.

### 3.4. Element Type and Mesh Size

Figure 8 presents a typical FE model of the NC, PUG, and NC-PUG composite beams. The NC and PUG were designed as three-dimensional eight-node composites with a reduced integration point (C3D8R) that was adopted for NC and PUG. The contact surface between NC and PUG used common mesh seeds to ensure accuracy. A sensitivity analysis was conducted with various mesh sizes, and the finest mesh with a size of 10 mm was selected according to the mesh convergence learning process.

### 3.5. Contact Modeling

The interaction between the NC and PUG grouting material was defined using surface-to-surface contact obtained in ABAQUS. The contact pair in the FE models consists of NC and PUG. The contact surface associated with the NC was used for the master surface, and the PUG contact surface was selected as the slave surface. Similarly, the contact surface associated with the NC-PUG composite was used as the master surface. A friction coefficient of 0.4 was used between the two components [47]. The normal contact behavior was assumed to be hard contact, allowing for the transmission of the surface’s pressure and separation when the pressure was zero or negative. The NC was designated as the host element.

### 3.6. Material Constitutive Model

The ABAQUS concrete damage plasticity (CDP) model simulated the NC and PUG’s material behavior. The CDP is a uniaxial compression and uniaxial tension plasticity model that describes the inelastic and damage behavior of concrete. The yielding criteria defined by Lee et al. [48] were adopted in this study. The concrete was characterized using the CDP model. The material properties of grade C50 concrete and PUG grouting material are summarized in Table 6.

### 3.7. Material Constitutive Model of NC

**Figure 9:**
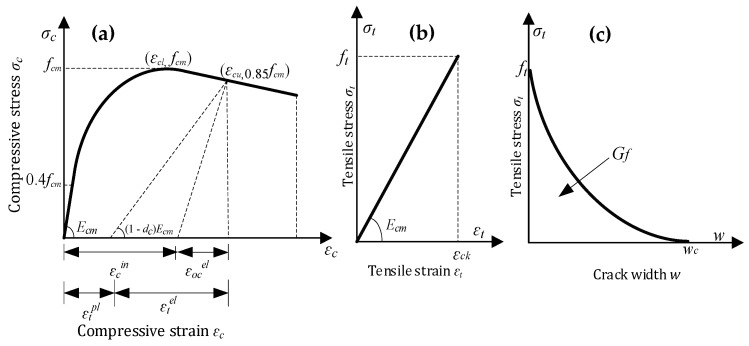
Concrete-damaged plasticity model of NC in (**a**) stress–strain in compression, (**b**) stress–strain in tension, and (**c**) stress–crack width.

The CDP model consisted of concrete compression and tensile damage; Figure 9 presents the stress–strain curves of the NC. The compressive stress–strain curve is classified into three parts. The first part is the elastic up to 0.4 *f*_mm_. The second section is the ascending parabolic part starting from 0.4 *f*_mm_ to *f*_mm_, which is calculated from Equation (2)—according to EC2—and the third part linearly descends from *f*_mm_ to 0.85 *f*_mm_.

(2) $$\begin{array}{l} \sigma_{c}=f_{cm}\left[\frac{kn-n^{2}}{1+(k-2)n}\right]\;\;\\ n=\frac{\varepsilon_{c}}{\varepsilon_{co}},\;k=E_{c}\varepsilon_{co}/f_{mm} \end{array}$$

where *Ɛ_c_* represents the compressive strain, *σ*_c_ represents the NSC compressive stress, *f_mm_* represents the ultimate compressive stress, *Ɛ_co_* represents the strain corresponding to *f_mm_* which is equal to 0.002, and the ultimate strain *Ɛ_cu_* is equal to 0.0035 [49]. *E*_c_ is the modulus of elasticity. Figure 1b shows the tensile stress–strain curve, in which the stress rises proportionately to the strain before cracking. The tensile strength (*f_t_*) is calculated using Equation (3) [50], and the equivalent strain (*Ɛ_ck_*) is defined as *f_t_*/*E*_c_.

(3) $$f_{t}=0.395f_{cu}^{0.55}$$

The relationship between tensile stress and cracking width is calculated using the fracture energy cracking model to define the tensile behavior of the NSC after cracking. The fracture was obtained from CEP-FIP [51] using Equation (4).

(4) $$G_{f}=G_{f10}{\left(\frac{f_{c}}{10}\right)}^{0.7}$$

where parameter *G*_f_ represents the fracture energy in Nmm/mm^2^, and the diameter of the coarse aggregate is approximately 16 mm. Hence, *G*_f10_ = 0.003 Nmm/mm^2^. The concrete compressive and damage coefficient, i.e., *d_c_* and *d_t_*, represents the damage behavior of the concrete. Thus, *d_c_* = 1 – *f_mm_*/*σ_c_* and *d_t_* = 1 – *f_t_*/*σ_t_*.

### 3.8. Model Validations

**Figure 10:**
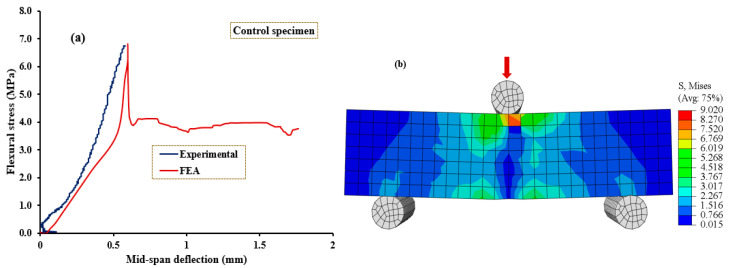
Validation of CDPM under flexure. (**a**) Numerical and experimental stress–strain models; (**b**) FEM and stress distribution for NC-PUG0.

**Figure 11:**
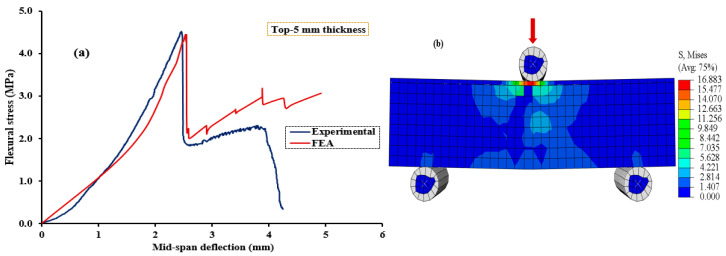
Validation of CDPM under flexure. (**a**) Numerical and experimental stress–strain models; (**b**) FEM and stress distribution for NC-PUGT5.

**Figure 12:**
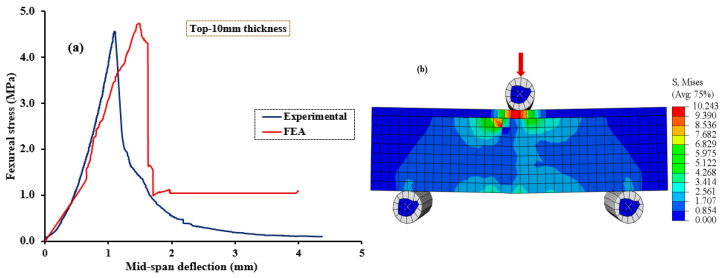
Validation of CDPM under flexure. (**a**) Numerical and experimental stress–strain models; (**b**) FEM and stress distribution for NC-PUGT10.

**Figure 13:**
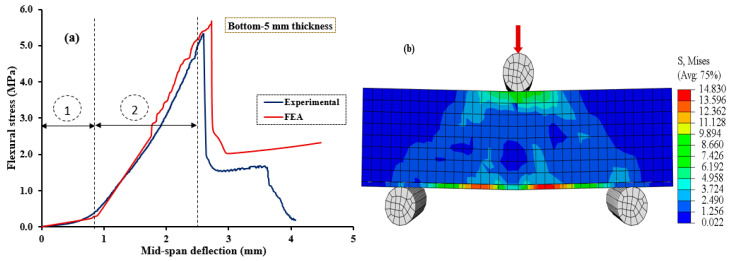
Validation of CDPM under flexure. (**a**) Numerical and experimental stress–strain models; (**b**) FEM and stress distribution for NC-PUGB5.

**Figure 14:**
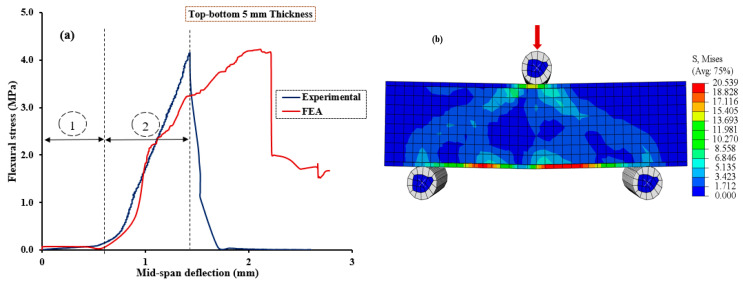
Validation of CDPM under flexure. (**a**) Numerical and experimental stress–strain models; (**b**) FEM and stress distribution for NC-PUGTB5.

Figure 10, Figure 11, Figure 12, Figure 13 and Figure 14 compare the flexural load–deflection curves between the experiments and the numerical model for each specimen condition. The FE analysis showed good agreement between the numerical model and the experimental test result. The initial stiffness (Ke) of the load–deflection curves between the numerical and experiment were compared for each specimen. Initial stiffness is explained as the ratio of 45% of the maximum load (P0.45) to the corresponding deflection (Δ0.45) as described by the ACI 318M-05 [52] and given in Equation (5).

(5) $$Ke=\frac{P_{0.45}}{\Delta_{0.45}}$$

The relationship between the flexural stress and mid-span deflection showed a linear pattern at the initial loading stage for the reference, and all the specimens were retrofitted at the top surface, as shown in Figure 10, Figure 11 and Figure 12, regardless of the PUG grout overlaid thickness. Common behaviors were observed for both the FE model and experimental test results. Under these conditions, deflection continuously increases with the applied loads until the specimen reaches the ultimate load and then fails in flexure. Nearly equal ultimate flexural stress was observed between the FE simulation and experimental test result.

On the other hand, two deformation regions were observed in the NC-PUG composite specimens strengthened with the PUG overlaid cast at the bottom surface or top-bottom surface, as revealed in Figure 13 and Figure 14, before complete failure. Under this configuration, polyurethane grout material was cast at the tension zone of the composite specimen, and polyurethane exhibited viscoelastic properties in response to the increased deflection without significance stress. This behavior tends to change the concrete’s brittle nature to a ductile state; polyurethane is a very strain rate-sensitive elastomer, with a significant change in performance from rubbery to leathery in response to increased strain rates. As a result, two deformation regions occurred during testing. Region (i): the initial loading condition; the relationship between flexural stress and deflection exhibits a flat slop with a drastic increase in mid-span deflection under continuous loading. The magnitude of the applied load became noticeable when the deflection reached a significant level, which marked the second deformation region. Region (ii): under this region, the relationship between the flexural stress and mid-span deflection exhibits a sharp slope. Both the applied load and deflection are increased rapidly until the test specimen fails in flexure. The two deformation regions demonstrated by these test specimens are caused by the viscoelastic behavior of polyurethane subjected to tensile stress at the tension zone of the composite specimens.

**Figure 15:**
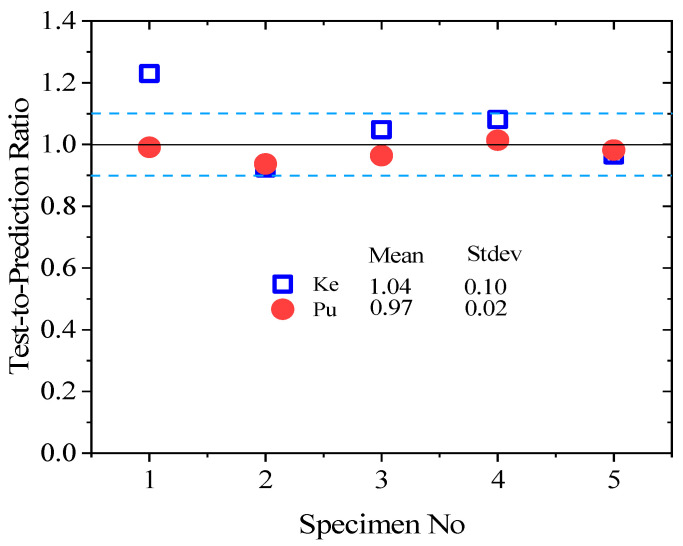
Scatter of test-to-prediction ratios for *K*_e_ and *P*_u_.

Table 7 presents the observed initial stiffness (Ke) and ultimate flexural stress (Pu) with numerical values, and the experimental test result-to-predicted value ratio of Ke and Pu is depicted in Figure 15. Table 7 and Figure 15 show that the FE model had accurately estimated the flexural responses of the NC-PUG beam and slightly underestimated Ke by 4% and overestimated Pu by 3%. The standard deviation of the flexural strength of the test specimen-to-prediction ratios of Ke and Pu are 0.1 and 0.02, respectively. The results indicated that the developed FE model predicts the flexural behavior and elastic stiffness of the NC-PUG specimens more accurately. It indicates that 80% of the predictions are within the range of ±10% of the prediction, as shown in Figure 15.

## 4. Conclusions

This study investigated the flexural behavior of the NC specimens retrofitted with a polyurethane grouting material of different thicknesses and configurations. The concrete beam specimens were subjected to a three-point bending test. The composite NC-PUG specimens were formed by casting the polyurethane grouting material at either top and/or bottom or both top-bottom surfaces. Moreover, finite element models were developed to simulate the flexural response of the NC-PUG specimens. The following conclusion can be drawn to summarize the findings.

The reference specimen (NC-PUG0) showed the highest average ultimate flexural stress of 5.56 MPa ± 2.57% at a 95% confidence interval with a corresponding mid-span deflection of 0.49 mm ± 13.60%. However, due to the strengthened effect of the polyurethane grout, the deflection of the composite specimen was significantly improved.

1. The configuration and/or position of the PU grout material cast influenced the relationship between the flexural stress and mid-span deflection. Specimens retrofitted at the bottom surface exhibit two deformation regions.
2. The effect of the PU grouting material changes the brittle nature of concrete to a more ductile state due to the viscoelastic behavior of polyurethane. This behavior is more effective on the specimen retrofitted at the bottom surface.
3. The FE analysis showed good agreement between the numerical model and the experimental test result. The numerical model accurately predicted the flexural strength of the NC-PUG beam, slightly underestimating *K*_e_ by 4% and overestimating P_u_ by 3%.

## Figures and Tables

**Table 1:** Cement chemical composition.

Material	Oxides									
	SiO_2_	Al_2_O_3_	Fe_2_O_3_	CaO	MgO	K_2_O	Na_2_O	SO_3_	TiO_2_	LOI
Cement	23.27	4.41	2.45	62.85	1.42	0.48	0.21	2.57	0.08	1.82

**Table 2:** Physical and performance parameter of PU binder.

PU Materials	Viscosity (CPS)	Appearance	Curing Age (h)		Tension Property (MPa)
			Initial	Final	
Castor oil	35,000	Grey/white sticky	-	-	-
PAPI	250	Brown transparent	-	-	-
PU binder	-	-	3.5	72	5.5

**Table 3:** NC and PUG design mix.

Mix ID	Cement (kg/m^3^)	Sand (kg/m^3^)	Coarse aggregate (kg/m^3^)	Water (kg/m^3^)
NC	425	718	966	170
	PU/Quartz sand (weight ratio)	PU binder (200 g)		
		Castor oil (g)	PAPI (g)	Solvent (g)
PU grout	1:0.5	167	33	8.4

**Table 4:** The NC-PUG configuration.

Sample ID	Sample Type	Sample Designation	PU Grout Layer Thickness (mm)	
			Top Surface	Bottom Surface
NC-PUG0	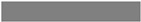	-	-	-
NC-PUGT5	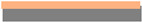	T	5	-
NC-PUGB5	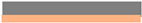	B	-	5
NC-PUGTB5	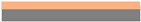	T&B	5	5
NC-PUGT10	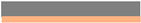	T	10	-

**Table 5:** The flexural response of concrete retrofitted with PU grout material.

Specimen ID	Code	Flexural Strength (MPa)	L-Deflection (mm)	Max Deflection (mm)	R-Deflection (mm)
Reference	1	5.478	0.42	0.57	0.42
	2	5.746	0.36	0.43	0.34
	3	5.478	0.35	0.47	0.38
	Confidential level (0.95)	5.56 ± 2.57%	0.38 ± 9.29%	0.49 ± 13.60%	0.38 ± 9.73%
NC-PUGB5	1	4.371	2.07	2.46	2.71
	2	4.32	2.2	2.60	2.43
	3	4.21	2.32	2.56	2.30
	Confidential level (0.95)	4.30 ± 1.77%	2.19 ± 5.26%	2.54 ± 2.62%	2.48 ± 7.81%
NC-PUGT5	1	3.662	2.32	2.46	2.42
	2	3.174	1.73	1.81	1.71
	3	3.330	2.24	2.22	2.21
	Confidential level (0.95)	3.39 ± 6.80%	2.09 ± 14.10%	2.16 ± 14.04%	2.11 ± 15.95%
NC-PUGT10	1	3.723	1.35	1.34	1.24
	2	3.702	0.97	1.1	1.04
	3	3.46	1.14	1.36	1.21
	Confidential level (0.95)	3.63 ± 3.72%	1.15 ± 15.25%	1.27 ± 10.55%	1.16 ± 8.57%
NC-PUGTB5	1	4.440	1.38	1.48	1.27
	2	4.155	1.43	1.2	1.28
	3	4.67	1.40	1.45	1.39
	Confidential level (0.95)	4.35 ± 3.62%	1.40 ± 1.66%	1.38 ± 10.32%	1.31 ± 4.68%

**Table 6:** Mechanical characteristics of NC and PUG.

Material	Compressive Strength (MPa)	Elastic Modulus (GPa)	Tensile Strength (MPa)	Density (kg/m^3^)
NC (C50)	48.67	32.29	4.76	2400
PUG	19.89	36.67	14.29	2400

**Table 7:** Comparison of experimental results with FE model predictions.

No.	Specimens	*K*e kN/mm	*K*_N_ kN/mm	*P*_u_ (kN)	*P*_N_ (kN)	*K*e/*K*_N_	*P*_u_/*P*_N_
1	NC	22.880	18.511	18.260	18.427	1.230	0.991
2	NC-PUGB5	3.661	3.963	14.400	15.365	0.924	0.937
3	NC-PUGT5	7.914	7.585	12.340	12.788	1.048	0.964
4	NC-PUGT10	3.541	3.275	12.210	12.034	1.081	1.014
5	NC-PUGBTB5	6.038	6.245	13.850	14.100	0.966	0.982
	Standard deviation	7.221	5.519	2.194	2.251	0.106	0.026
	Mean	8.807	7.916	14.212	14.543	1.049	0.977
	Cov (%)	82	69.7	15.4	15.5	10.1	2.6

## Data Availability

The data presented in this study are available on request from the corresponding author.
